# Effects of psychotropic switches on weight change: a prospective cohort study

**DOI:** 10.1192/j.eurpsy.2022.1887

**Published:** 2022-09-01

**Authors:** M. Piras, S. Ranjbar, C. Dubath, N. Laaboub, C. Grosu, F. Gamma, K. Von Plessen, A. Von Gunten, P. Conus, C. Eap

**Affiliations:** 1 Lausanne University Hospital, Psychiatry, Prilly, Switzerland; 2 Les Toises Psychiatry and Psychotherapy Center, Psychiatry, Lausanne, Switzerland

**Keywords:** psychopharmacology, Weight gain

## Abstract

**Introduction:**

Many psychotropic drugs can induce weight gain with differences in their metabolic risk profiles (i.e. high, medium or low-risk).

**Objectives:**

To compare the weight evolution of patients switching versus patients keeping their psychotropic drugs with different risk-profiles.

**Methods:**

Data for patients switching or keeping the same drug were obtained from the Psyclin (from 2007 to 2015) and Psymetab (2007- 2019) cohort studies, conducted at the Lausanne University Hospital, Switzerland. Patients either switched from a high to a low-risk, a high to a medium-risk, a medium to a low-risk drug, or for a drug with the same risk category. Patients not switching either kept a high, medium or low-risk drug. The evolution of weight is currently being analyzed using a linear mixed-effect model.

**Results:**

Preliminary results showed that switching from a high to low-risk molecule had the strongest impact on weight changes. The analysis being ongoing, the quantitative results will be presented at the congress.

**Conclusions:**

Switching from a high-risk to a low-risk molecule is likely to have the strongest impact on weight changes.

**Disclosure:**

No significant relationships.

